# Performance of an automated chemiluminescent immunoassay for SARS-COV-2 IgM and head-to-head comparison of Abbott and Roche COVID-19 antibody assays

**DOI:** 10.1016/j.plabm.2021.e00230

**Published:** 2021-04-28

**Authors:** Lau CS, Hoo SP, Liang YL, Phua SK, Aw TC

**Affiliations:** a Department of Laboratory Medicine, Changi General Hospital, Singapore; b Department of Medicine, National University of Singapore, Singapore; c Academic Pathology Program, Duke-NUS Medical School, Singapore

**Keywords:** SARS-CoV-2, Antibodies, Assay evaluation, IgM

## Abstract

**Introduction:**

We evaluated the performance of the new Abbott SARS-CoV-2 IgM assay on the Architect immunoassay analyser and compared it to the Architect IgG/Roche Cobas total antibody assays in both SARS-CoV-2 RT-PCR positive cases and healthy controls.

**Method:**

200 healthy control samples and 48 individuals with other antibody-positive disorders (18 hepatitis/18 dengue/11 ANA/1 dsDNA) served to assess for potential cross-reactivity. Anonymised residual leftover sera positive for SARS-CoV-2 on RT-PCR were recruited as cases (N ​= ​133). The sensitivity/specificity/cross-reactivity of the Architect IgM assay were assessed. Concordance between the 3 assays were also analysed.

**Results:**

There was no cross-reactivity with controls and other antibody positive samples. The Architect IgM assay was 100% specific (95% CI 98.5 to 100) and sensitivity was 77.8% (95% CI 60.8 to 89.9) ≥14 days post-first positive RT-PCR (POS). Sensitivity of the combined Architect IgM and IgG results (30.8%) was significantly better than the Cobas total antibodies (15.4%) in early disease (p ​= ​0.04). While the Architect IgM assay had moderate agreement with the Cobas total antibody result (Cohen’s kappa 0.72), a combined Architect IgM and IgG result had better agreement (Cohen’s kappa 0.83).

**Conclusion:**

The Architect IgM assay has good specificity and no cross-reactivity with other antibody positive cases. A combined Architect IgM and IgG result has better sensitivity than the individual assays for early COVID-19. The Architect IgM assay is not comparable to the Cobas total antibody assay, but the Architect IgM and IgG combined result has good agreement with the Cobas assay.

## Highlights

• IgM/IgG may not develop in some cases of COVID-19, even ≥14 days post infection.
• The sensitivity/PPV of the combined Architect IgG/IgM assays improves ≥14 days POS.
• The Architect IgM/IgG combined result has good agreement with the Cobas assay.
• The Architect IgM and IgG combined results slightly outperforms the Cobas assay.

## Abbreviations

SARS-CoV-2: Novel severe acute respiratory syndrome coronavirus 2
COVID-19: Coronavirus disease 2019
RT-PCR: Reverse-transcriptase polymerase chain reaction
POS: Post-first positive RT-PCR
HS: health screening
ANA: anti-nuclear antibody
ds-DNA: double-stranded DNA antibody
COI: Cut-off index
PPV: Positive predictive value
NPV: Negative predictive value

## 1 Introduction

Although reverse-transcriptase polymerase chain reaction (RT-PCR) testing remains the recommended diagnostic test for novel severe acute respiratory syndrome coronavirus 2 (SARS-CoV-2) infection, up to 13% of patients can have low viral loads with negative RT-PCR tests [1]. RT-PCR testing only has a sensitivity of around 79% at best [2], with a false-negative rate of 38% on the day of disease onset, decreasing to 20% on day 8 of disease onset [3]. The US Centers for Disease Control and Prevention recommends serologic assays for use in monitoring the pandemic [4] and in suspected coronavirus disease 2019 (COVID-19) cases with negative RT-PCR, and the Infectious Diseases Society of America recommended that serologic testing could be used in patients with a high clinical suspicion for COVID-19 but with negative RT-PCR results two weeks post-symptom onset and for sero-surveillance studies [5]. Serology thus has a role as a possible adjunct to RT-PCR testing. IgM levels can rise as early as day 5 post disease onset in patients with mild disease [6] and increase significantly in patients with severe COVID-19 [7]. It is also possible for patients or close-contacts who are RT-PCR negative to have virus-specific IgM in initial samples [8].

We have previously evaluated the Abbott SARS-CoV-2 IgG antibody [9] and the Roche total SARS-CoV-2 total antibody assays [10], run on the Architect i2000 and Cobas e801 immunoassay analysers respectively. These two assays have excellent performance. Abbott has recently released a new SARS-CoV-2 IgM assay for the Architect analyser, and there is paucity of data on the performance of this new assay. SARS-CoV-2 IgM detection may be of use in the identification of early COVID-19. As such, we evaluated the performance of the new Architect SARS-CoV-2 IgM assay and compared it to the Architect IgG and Cobas total antibody assays in SARS-CoV-2 RT-PCR positive subjects and COVID-19 naive cases.

## 2 Methods

**Fig. 1:**
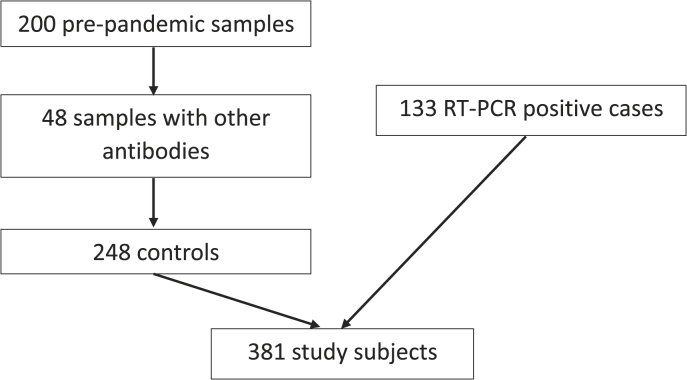
Flow chart detailing the composition of the study population. *Abbreviations: RT-PCR: Reverse-transcriptase polymerase chain reaction.*

**Participants:** Residual leftover sera were used in this study. Two-hundred pre-pandemic samples from a staff health screening (HS) program in 2018 served as controls. In addition, 48 pre-pandemic/current antibody positive samples (18 viral hepatitis [B or C or E] (taken in 2020), 18 dengue (taken in 2020), 11 anti-nuclear antibody [ANA] and 1 double-stranded-DNA antibody [dsDNA] (pre-pandemic)) were used to assess for potential cross-reactivity. All pre-pandemic samples were non-reactive on the Architect IgG and Cobas assays, and all cross-reactivity samples taken in 2020 had COIs comparable to pre-pandemic samples, establishing that they were likely to be free of COVID-19. Residual de-identified sera from other routine laboratory testing (e.g. renal panels, blood cell counts) in subjects who tested positive for SARS-CoV-2 on RT-PCR from April to June 2020 were recruited as cases (N ​= ​133) (see Fig. 1). 78 RT-PCR positive samples were repeat sample from 27 patients. All samples were stored at −70 degrees Celcius, being freeze-thawed only once before. Days post-first positive RT-PCR (POS) was used as a surrogate for disease onset, and results stratified according to days POS. Demographics of the cases and control subjects are displayed in Table 1. As this work involved de-identified leftover sera and was part of evaluating new diagnostic assays and seroprevalence surveillance, it was deemed exempt by our institutional review board.

**Table 1:** Population demographics.

	N	Age (mean ​± ​SD)	Males (%)	Females (%)
Cases	133	26 to 98 (51.0 ​± ​17.7)	108 (81.2)	25 (18.8)
Controls	248	25 to 85 (47.2 ​± ​12.7)	49 (20.9)	185 (79.1)

Abbreviations: SD: standard deviation.

**Instrumentation and materials:** The Abbott SARS-CoV-2 IgG assay is a qualitative chemiluminescent microparticle immunoassay run on the ARCHITECT i2000 System, as described previously [9]. The stated cut-off index (COI) of the Architect IgG assay is 1.4. In our own evaluation, the assay sensitivity for samples ≥14 days POS is 96.7%, and for samples <7 days is 45.9% [9]. The Roche anti-SARS-CoV-2 assay is a non-competitive sandwich immunoassay, also described previously [10], with a stated COI of 1.0. In our own evaluation [10], the assay specificity was 99.9%, and the assay sensitivity was 97.1% when ≥14 days POS and 48.2% when <7 days POS. Both the Abbott IgG assay and Roche total antibody assay target antibodies directed against the viral nucleocapsid. For RT-PCR testing, our hospital molecular laboratory employs a duplex real-time RT-PCR that targets the N and E genes using a Qiagen EZ1 extraction system and Rotor Gene Q amplification system.

The Architect SARS-CoV-2 IgM antibody assay also utilizes a chemiluminescent microparticle immunoassay similar to the Architect IgG assay but targets the IgM antibodies directed against the spike antigens on the virion. In a chemiluminescent reaction, relative light units directly proportional to the amount of SARS-CoV-2 IgM titres is reflected as a COI; a COI above 1.0 connotes reactivity. The manufacturer reported precision of the assay (CV) is 2.8 (at COI 1.96) and 2.9% (at COI 2.80), with a reported sensitivity of 59.5% (0–7 days POS) to 100% (>14 days POS) and a specificity of 99.6% in the package insert.

**Statistical analysis:** For precision analysis, negative and positive Abbott controls were run 10 times each. As the Architect IgM assay is a qualitative test, the diagnostic specificity of the test is represented by the negative percentage agreement between antibody negativity against all control subjects; diagnostic sensitivity is represented by the positive percentage agreement between antibody positivity against all RT-PCR positive patients. We also performed a concordance analysis between the Architect IgG, Architect IgM and the Cobas total antibody results, and attempted to calculate the positive predictive value (PPV) and negative predictive value (NPV). No data with indeterminate or missing results were used.

Data were presented in either mean ​± ​standard deviation or median [inter-quartile range], as appropriate. 95% confidence intervals for sensitivity and specificity were calculated according to Clopper and Pearson exact method with standard logit confidence intervals for predictive values. Between-test agreements were assessed by interpretation of Cohen’s Kappa-statistics. Statistical analyses were performed using MedCalc® Statistical Software version 19.5.3 (MedCalc Software Ltd, Ostend, Belgium). For the 95% confidence interval in groups with 100% PPV, we used Stata 14 software (StataCorp. 2015. Stata Statistical Software: Release 14. College Station, TX: StataCorp LP). Compliance with STARD guidelines is enclosed (see Supplementary Table 1).

## 3 Results

**Precision:** For precision analysis, negative and positive Abbott control material was run 10 times each. For negative controls the within run CV was 21.0% (at COI 0.02), and for positive controls the within run CV was 2.4% (at COI 2.99); between run CV was 15.5% (at COI 0.03) and 3.7% (at COI 2.30) respectively.

**Cross-reactivity and specificity:** All 48 samples positive for hepatitis, dengue, ANA and dsDNA antibodies showed no cross-reactivity with the Architect IgM assay. All 248 controls and cross-reactivity samples tested negative on the Architect IgM assay, with a specificity of 100% (95% CI 98.5 to 100).

**Sensitivity:** The Architect IgM assay sensitivity increased with days POS. However, the sensitivity remained at 77.8% even after 14 days POS (see Table 2). 48.9% (n ​= ​65) of cases did not develop an Architect IgM signal even after 14 days, and 49.6% (n ​= ​66) of cases did not develop an Architect IgG signal after 14 days. In cases tested 0–6 days POS (early disease), the Architect IgM had a sensitivity of 26.2% while the Architect IgG had a sensitivity of 10.8%.

**Table 2:** Sensitivity of the Architect IgM vs the Architect IgG and Roche total antibody assays.

Days POS	Architect IgM			Architect IgG			Either or both Architect IgM and IgG			Roche total antibody		
	Positive	Negative	Sensitivity (95% CI)	Positive	Negative	Sensitivity (95% CI)	Positive	Negative	Sensitivity (95% CI)	Positive	Negative	Sensitivity (95% CI)
0 to 6	17	48	26.2	7	58	10.8	20	45	30.8	10	55	15.4
			(16.0–38.5)			(4.44–20.9)			(19.9–43.4)			(7.63–26.5)
7 to 13	23	9	71.9	25	7	78.1	25	7	78.1	25	7	78.1
			(53.3–86.3)			(60.0–90.7)			(60.0–90.7)			(60.0–90.7)
≥14	28	8	77.8	35	1	97.2	36	0	100	35	1	97.2
			(60.8–89.9)			(85.5–99.9)			(90.3–100)			(85.5–99.9)
Total	68	65	51.1	67	66	50.4	81	52	60.9	70	63	52.6
			(42.3–59.9)			(41.6–59.2)			(52.1–69.2)			(43.8–61.3)

Abbreviations: POS: post-first positive RT-PCR, CI: Confidence interval.

**Fig. 2:**
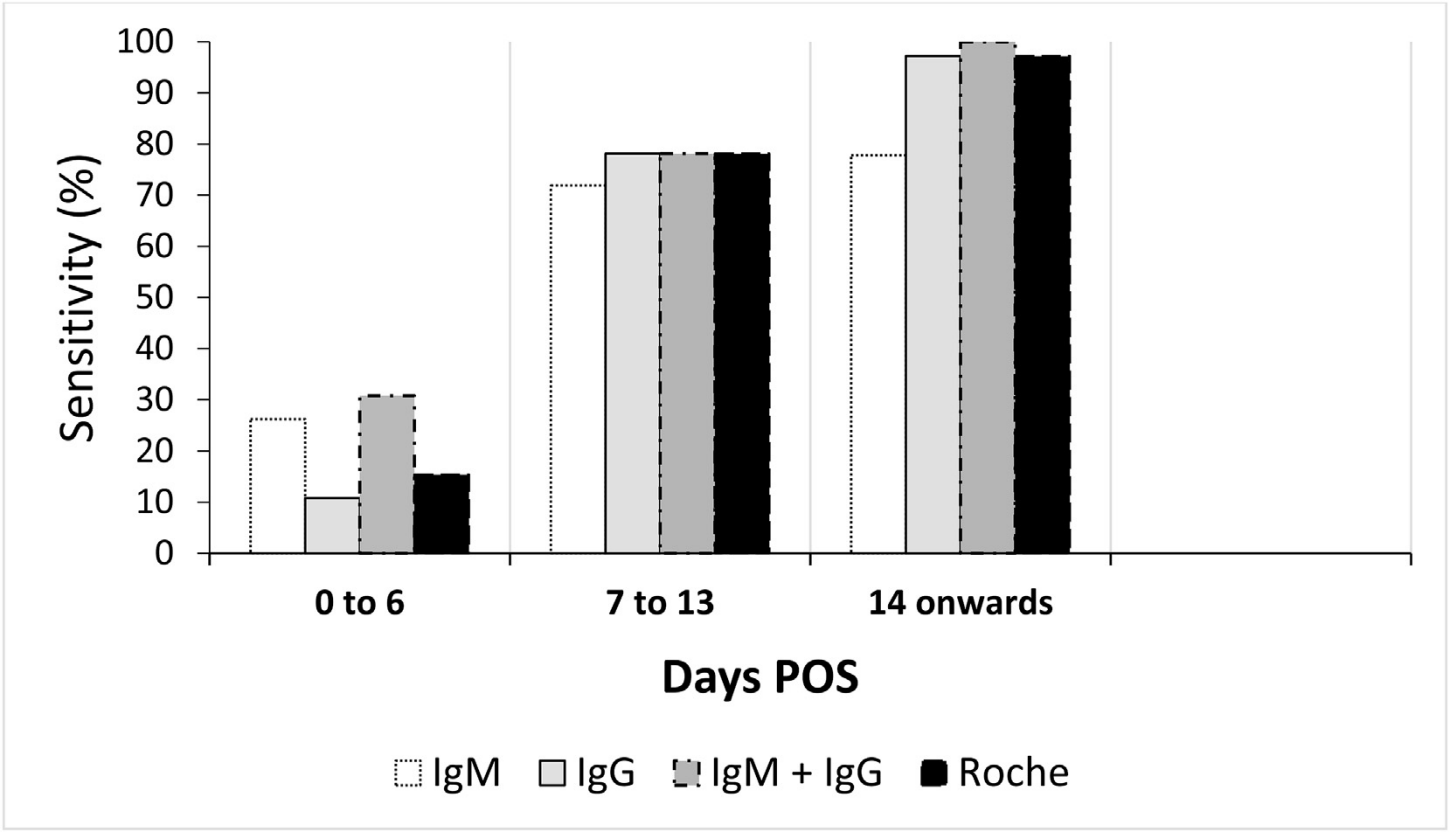
Progression of sensitivity with days POS of Architect IgM, IgG, IgM and IgG, and Roche Cobas total antibody assays. *Abbreviations: POS: post-first positive RT-PCR.*

When both Architect IgM and IgG results were interpreted together, any reactive IgG and/or IgM result was considered as positive for COVID-19. Only 39.1% (n ​= ​52) of cases were negative after 14 days (see Table 2). The combined Architect IgM and IgG result also had a similar sensitivity to the Architect IgG and Cobas assays at ≥14 days POS (100% vs 97.2% and 97.2%). The Cobas assay sensitivity was 15.4% in days 0–6 days POS. However, the combined Architect IgM and IgG results had better sensitivity of 30.8% (95% CI 19.9 to 43.4) for 0–6 days POS. The trend observed was that for all assays, sensitivity generally increased with days POS (see Fig. 2), however, only the Architect IgG, the combined Architect result, and the Roche total antibody assays achieved >95% sensitivity after 14 days POS.

**Concordance:** Agreement between assays were studied in a pairwise fashion by applying inter-rater agreement statistics (Cohen Kappa). Individually, the Architect IgG had good agreement with the Cobas assay (kappa 0.88; 95% CI 0.82 to 0.95). The agreement between Architect IgM and Cobas assays was moderate (kappa 0.72; 95% CI 0.62 to 0.81) but improved when combining the Architect IgM and IgG assays (kappa 0.83, 95% 0.76 to 0.90), with 94.5% (n ​= ​360) concordant cases.

## 4 Discussion

On its own, the Architect IgM assay has moderate sensitivity (26.2%) for antibody detection within the first week of infection, increasing in the second week to 71.9% and peaks at 77.8% thereafter. This is in keeping with other studies [11], where IgM levels peak in the second week after symptom onset and may even decline by the third week. In another study the sensitivity of the Abbott IgM assay from positive PCR results was 63.6% at day 0 (n ​= ​22), 76.5% at day1–5 (n ​= ​17), 76.3% at day 6–14 (n ​= ​38), 85.2% at day 15–30 (n ​= ​54), and 63.6% over 30 days (n ​= ​44) [12]. However, the best value of the Architect IgM is in antibody detection in early disease [12], where we noted its higher sensitivity compared to the Cobas assay (15.4%). This is also supported by other studies in larger cohorts [13]. In a study of 972 hospitalized patients and 586 normal volunteers, the sensitivity of IgM was 82.54% in the first week of infection, compared with 80.95% with IgG. In another study evaluating the Abbott SARS-CoV-2 IgM and IgG assays in 300 control samples and 1349 sequential samples from 427 RT-PCR positive patients [14], the clinical test sensitivity of the IgM and IgG assay was 24.6% and 23.2% in the first week of infection, and the positive rate was 30.8% and 92.3% at >3 months later.

The sensitivity of the Architect IgM in early disease is further improved by combining it with the Architect IgG results. For cases at 0–6 days POS, this combined result increases the sensitivity of antibody detection from 26.2% to 30.8%, exceeding that of the Cobas total antibody (15.4%) and Abbott IgG (10.8%) assays. The sensitivity of the Cobas total antibody assay was marginally higher than the individual Architect IgG and IgM results in the total population. Furthermore, the sensitivity of the combined Architect IgM and IgG results exceeded 95% after 14 days POS. Other studies [8] have also reported that a combined assessment of IgG and IgM (with positive IgG and/or IgM considered as reactive) offers a higher sensitivity than testing for either IgG or IgM alone in the early stages of COVID-19. A recent Cochrane review [15] reported that the combination of IgG and IgM had a sensitivity of 30.1% within the first week of COVID-19, rising to 72.2% by the second week and 91.4% after that. The Cochrane review noted that individual IgG and IgM testing had a low sensitivity <30.1% in the first week of infection. The findings of the Cochrane review are fairly similar to the progression of the combined Architect IgG and IgM sensitivity found in our study. Differences between the combined Abbott IgG with IgM result and the Cobas total antibody sensitivities can be attributed to differences in assay design: the Abbott IgM assay targets spike-antibodies, whereas the Roche total antibody and Abbott IgG assays target nucleocapsid antibodies. There is some evidence that spike assays may have increased sensitivity compared to nucleocapsid assays [16].

There is a clear concordance between the Architect IgG and Cobas total antibody assays. One study [17] comparing the Abbott IgG and Cobas antibody assays showed a Kappa of 0.87, which is similar to what we found (0.88). In our study, the Architect IgM had a more modest agreement with the Cobas assay (kappa 0.72); the combined Architect IgG and IgM results agreed well with the Cobas assay (kappa 0.83).

Some studies show that IgG can be more sensitive in early disease than IgM (IgG 64.7% vs IgM 38.2% sensitivity in days 5–10 after disease onset) [18]. Others [19] report that IgM has an earlier seroconversion (18 days) compared to IgG (20 days). In our study, out of the 81 positive cases using the combined result of both Architect IgG and IgM, 16.0% (n ​= ​13) were IgG positive but IgM negative, and 17.3% (n ​= ​14) were IgM positive but IgG negative. In one study of 17,368 subjects [20], IgG responses were earlier and higher than those of IgM. Liu et al. [21] also reported that up to 40% of COVID-19 patients can fail to develop an IgM response. This lends further support that a total antibody evaluation would be more appropriate at any point in the course of COVID-19 infection as previously alluded in the Cochrane review [15].

The strengths of our study are that we have assessed the sensitivity performance of the Architect IgM assay stratified by groups of days POS. We also confirm that dengue antibodies have no cross-reactivity with the Architect IgM antibody assay, as some studies [22] have shown that dengue infections can cause false positive reactivity with some COVID-19 assays.

One limitation of our study is that it is comprised of a hospital population. The serological status was compared to the timing of RT-PCR positivity and not relative to timing of symptom onset. We are thus unable to account for any delays between start of infection and RT-PCR. However, the use of RT-PCR as a surrogate for disease onset would include subjects who are possibly asymptomatic (as we do not have the clinical history of the deidentified samples). The proportion of males is substantially higher in our cases, as this reflects the real-world male preponderance of more severe COVID-19 [23]. The higher proportion of females in our control population will not have affected our analysis, as all their COIs were below the LOD. In the general population, SARS-CoV-2 antibody responses might be lower due to a higher number of asymptomatic and pauci-symptomatic infections [[24], [25], [26]]. In one study [27], 38 patients with severe COVID-19 were compared to 24 mild COVID-19 infections; 65% of severe cases demonstrated antibody activity, compared to 30% in mild cases. Liu et al. [21] showed a significant difference in the IgG response between patients with mild and severe infection. In some mild cases (21.43%), IgG antibodies did not develop until 9 days after symptom onset. Thus, assay sensitivity, specificity, PPV and NPV might be affected by differences in antibody titres and disease prevalence between hospital and general populations. The sensitivities of the Architect IgG and Cobas total antibody assays are lower than that in our previous evaluations [9,10]. This is because the population of RT-PCR positive cases in this evaluation is different (e.g. age and gender) and smaller than our previous studies (133 cases in the present evaluation compared to 349 cases for Cobas and 279 cases for Architect IgG). Another limitation is that our study showed that the assay had 100% specificity which would lead to overly optimistic PPV (100%), as shown in Supplementary Table 2. This may be due to our study having too few controls, with a larger proportion of controls coming from healthy volunteers, rather than more samples with cross-reacting antibodies. The manufacturer reported assay specificity is 99.6%, as they used a large population of 2965 controls, including subjects with various conditions such as hemodialysis, influenza, lupus and other respiratory viruses. A specificity of 99.6% would result in a PPV of 86.0% with all our cases (68 positive and 65 nega tive cases on the Architect IgM) if the disease prevalence were 5%. We also observed that, if the specificity were not 100%, the PPV would evolve with days POS (0–6 days POS 75.8%, ≥14 days POS 90.3%). The difference in PPV and NPV is further exacerbated in populations with lower disease prevalence, as the PPV would naturally decrease with prevalence and the NPV would exceed its already high value. However, with a 100% PPV, the predictive value remains static and does not change with days POS or disease prevalence. As such, further evaluations of the Architect IgM assay involving a larger number of subjects would be desirable. The FDA also recommends that it is important to assess cross-reactivity not only with viral hepatitis, but also other corona viruses, influenza and respiratory syncytial virus, and to obtain a minimum of 5 individual samples for each antibody class [27]. We had no access to such samples. However, the manufacturer and other publications [[28], [29]] have explored cross-reactivity with other corona viruses extensively, and report little evidence of cross-reactivity.

## 5 Conclusion

In summary, we report some new findings for the new Architect IgM assay. The IgM or IgG may not develop in some cases of COVID-19 infection, even after 14 days POS. The Abbott IgG and IgM results should be interpreted together. Either IgG and/or IgM positivity should be considered as positive for seroconversion. A negative IgM or IgG result does not exclude COVID-19 infection. The sensitivity and PPV of the combined Architect IgG and IgM assays improve after 14 days POS, and even outperforms the Cobas total antibody assay.

## Disclosures

All co-authors have contributed to the study and manuscript.

## Declaration of competing interest

The authors declare that they have no known competing financial interests or personal relationships that could have appeared to influence the work reported in this paper.
