# Supplementary material for: Performance of an automated chemiluminescent immunoassay for SARS-COV-2 IgM and head-to-head comparison of Abbott and Roche COVID-19 antibody assays
Source: Pract Lab Med. 2021 Apr 28;25:e00230. doi: 10.1016/j.plabm.2021.e00230 (PMC8079267; doi:10.1016/j.plabm.2021.e00230)
Supplement: Multimedia component 1 [file mmc1.docx]

**Supplementary Table 1: STARD guideline checklist.**

|  | **Section & Topic** | **No** | **Item** | **Reported on page #** |
| --- | --- | --- | --- | --- |
|  |  |  |  |  |
|  | **TITLE OR ABSTRACT** |  |  |  |
|  |  | **1** | Identification as a study of diagnostic accuracy using at least one measure of accuracy  (such as sensitivity, specificity, predictive values, or AUC) | **2** |
|  | **ABSTRACT** |  |  |  |
|  |  | **2** | Structured summary of study design, methods, results, and conclusions  (for specific guidance, see STARD for Abstracts) | **2** |
|  | **INTRODUCTION** |  |  |  |
|  |  | **3** | Scientific and clinical background, including the intended use and clinical role of the index test | **4** |
|  |  | **4** | Study objectives and hypotheses | **4** |
|  | **METHODS** |  |  |  |
|  | *Study design* | **5** | Whether data collection was planned before the index test and reference standard were performed (prospective study) or after (retrospective study) | **5** |
|  | *Participants* | **6** | Eligibility criteria | **5** |
|  |  | **7** | On what basis potentially eligible participants were identified  (such as symptoms, results from previous tests, inclusion in registry) | **5** |
|  |  | **8** | Where and when potentially eligible participants were identified (setting, location and dates) | **5** |
|  |  | **9** | Whether participants formed a consecutive, random or convenience series | **5** |
|  | *Test methods* | **10a** | Index test, in sufficient detail to allow replication | **6** |
|  |  | **10b** | Reference standard, in sufficient detail to allow replication | **6** |
|  |  | **11** | Rationale for choosing the reference standard (if alternatives exist) | **6** |
|  |  | **12a** | Definition of and rationale for test positivity cut-offs or result categories  of the index test, distinguishing pre-specified from exploratory | **6** |
|  |  | **12b** | Definition of and rationale for test positivity cut-offs or result categories  of the reference standard, distinguishing pre-specified from exploratory | **6** |
|  |  | **13a** | Whether clinical information and reference standard results were available  to the performers/readers of the index test | **5** |
|  |  | **13b** | Whether clinical information and index test results were available  to the assessors of the reference standard | **5** |
|  | *Analysis* | **14** | Methods for estimating or comparing measures of diagnostic accuracy | **7** |
|  |  | **15** | How indeterminate index test or reference standard results were handled | **7** |
|  |  | **16** | How missing data on the index test and reference standard were handled | **7** |
|  |  | **17** | Any analyses of variability in diagnostic accuracy, distinguishing pre-specified from exploratory | **7** |
|  |  | **18** | Intended sample size and how it was determined | **Not applicable** |
|  | **RESULTS** |  |  |  |
|  | *Participants* | **19** | Flow of participants, using a diagram | **6** |
|  |  | **20** | Baseline demographic and clinical characteristics of participants | **5** |
|  |  | **21a** | Distribution of severity of disease in those with the target condition | **Not applicable** |
|  |  | **21b** | Distribution of alternative diagnoses in those without the target condition | **Not applicable** |
|  |  | **22** | Time interval and any clinical interventions between index test and reference standard | **Not applicable** |
|  | *Test results* | **23** | Cross tabulation of the index test results (or their distribution)  by the results of the reference standard | **8, 9** |
|  |  | **24** | Estimates of diagnostic accuracy and their precision (such as 95% confidence intervals) | **8** |
|  |  | **25** | Any adverse events from performing the index test or the reference standard | **Not applicable** |
|  | **DISCUSSION** |  |  |  |
|  |  | **26** | Study limitations, including sources of potential bias, statistical uncertainty, and generalisability | **12** |
|  |  | **27** | Implications for practice, including the intended use and clinical role of the index test | **14** |
|  | **OTHER INFORMATION** |  |  |  |
|  |  | **28** | Registration number and name of registry | **5** |
|  |  | **29** | Where the full study protocol can be accessed | **Not applicable** |
|  |  | **30** | Sources of funding and other support; role of funders | **15** |
|  |  |  |  |  |

**Supplementary Table 2: Comparison of the effect of specificity and disease prevalence on PPV/NPV.**

| Days POS | Specificity of 100% | | Specificity of 99.6% | |
| --- | --- | --- | --- | --- |
|  | PPV (95% CI) | NPV (95% CI) | PPV (95% CI) | NPV (95% CI) |
| Disease prevalence 5% | | | | |
| 0 to 6 | 100 (94.7 to 100) | 96.3 (95.7 to 96.7) | 75.8 (61.4 to 86.1) | 96.2 (95.7 to 96.7) |
| 7 to 13 | 100 (94.7 to 100) | 98.5 (97.5 to 99.2) | 89.6 (82.8 to 93.9) | 98.5 (97.5 to 99.2) |
| ≥14 | 100 (94.7 to 100) | 98.8 (97.9 to 99.4) | 90.3 (84.1 to 94.3) | 98.8 (97.9 to 99.4) |
| Total population | 100 (94.7 to 100) | 97.5 (97.0 to 97.9) | 86.0 (77.7 to 91.5) | 97.5 (97.0 to 97.9) |
| Disease prevalence 1% | | | | |
| 0 to 6 | 100 (94.7 to 100) | 99.3 (99.1 to 99.4) | 37.6 (23.4 to 54.3) | 99.3 (99.1 to 99.4) |
| 7 to 13 | 100 (94.7 to 100) | 99.7 (99.5 to 99.8) | 62.3 (48.0 to 74.8) | 99.7 (99.5 to 99.8) |
| ≥14 | 100 (94.7 to 100) | 99.8 (99.6 to 99.9) | 64.2 (50.3 to 76.0) | 99.8 (99.6 to 99.9) |
| Total population | 100 (94.7 to 100) | 99.5 (99.4 to 99.6) | 54.1 (40.0 to 67.5) | 99.5 (99.4 to 99.6) |

*Abbreviations: POS: post-first positive RT-PCR, PPV: positive predictive value, NPV: negative predictive value, CI: confidence interval.*
